# The Second Session

**Published:** 1856-05

**Authors:** 


### EDITORIAL AND MISCELLANEOUS.

### THE SECOND SESSION.

It has been already announced that the second course of lectures in the Atlanta Medical College would commence on the 1st day of May, and terminate on the last day of the following August, and we should not have made it the subject of farther remark, but for a report which has reached us, upon respectable authority, to the effect, that a certain individual connected with a medical school—during the course of lectures, the past winter, in that Institution—made an effort (how *openly* we have not learned) to produce the impression that the Atlanta Medical College was not recognised.

Now, we do not wish to repeat what has been already said, upon our rights and powers, and the ample recognition of our claims upon the part of others engaged in the same work, but intend to say, that we hope there has been some misunderstanding in this matter, as we are exceedingly reluctant to believe that there has been the willful and malignant misrepresentation of facts, which we would not hesitate to charge upon the author of such a statement.

We are, however, perfectly satisfied that there has been an attempt to prejudice the minds of students in reference to this Institution, and that in a manner which *we* cannot *recognise* as fair and honorable. We had supposed that the same code of ethics which all legitimate and regular medical men acknowledge as regulating their intercourse, as individual members of the medical profession—not to say as gentlemen—was equally applicable to the relation which we sustain to each other as medical teachers.

Indeed we had even thought that there was a more sacred obligation resting upon those who were expected to give the impress to young men, about entering the profession, to exhibit a freedom from the vice of selfishness, which is the besetting sin of mankind; and by appreciating and respecting the rights of others, to maintain the dignity and honor of our calling. And we had even thought that whatever *good policy* there might be in "aiming between the joints of the harness, when the blow was not looked for," it was *bad morality*, and that a triumph achieved, under such circumstances, would hardly afford much satisfaction to an honorable mind; but we are happy to know that the cowardly thrust has failed to reach our vitals, and that the life's blood is still coursing merrily on.

The cry of non-recognition has never been very alarming to us, knowing that our position was in every respect legitimate and proper; but if we had been disposed to apprehend any difficulty upon this point, we now announce it as an established fact beyond controversy, that the Atlanta Medical College has been awarded all, in this particular, that is necessary to place either our first course students or graduates upon the most honorable footing; for independent of other evidence entirely satisfactory, we have the *conclusive* testimony of our full recognition upon the part of the first medical colleges in the land, in the fact, that they have already admitted our students to a full participation in all the rights and immunities which belong to those who have attended a single course in their own institution.

In addition to this, we wish it distinctly understood, that we have never been *suppliants* for the favor or countenance of any; intending at the same time, of course, to conform to whatever requisitions might be established by the united action of the profession in body assembled; and further, we would inform those who may need the information, that "knowing our rights, we dare maintain them," in having a voice in the settlement of all the "vexed questions" which disturb the profession, or which may be sprung with a view of advancing its interests.

But not to prolong this subject, we would remark that any effort to discredit our claims and position is simply ridiculous, and could only have proceeded from a judgment blinded by morbid jealousy, which has so far only resulted in our benefit, and in this point of view, may be considered fortunate; though we would certainly prefer to rest upon our merits for advancement, having no taste or disposition for controversy, besides considering it discreditable to the profession; and anticipating as we do, a full measure of success, from the advantages which we hope to offer the medical student.

And to conclude, we are about entering upon another course of lectures, with most encouraging prospects, and a determined and united purpose to use every energy to sustain and increase the already prosperous condition of the Institution.

